# Lumbar Erector Spinae Plane Block for Dega Pelvic Osteotomy in a Pediatric Patient With Complex Neurological Issues: A Case Report

**DOI:** 10.7759/cureus.57263

**Published:** 2024-03-30

**Authors:** James A Hruschka, Pearl M Huynh, Timothy R Petersen, Stacey A Stallard, Codruta N Soneru

**Affiliations:** 1 Anesthesiology, Charleston Area Medical Center, Charleston, USA; 2 Anesthesiology and Critical Care Medicine, University of New Mexico School of Medicine, Albuquerque, USA; 3 Anesthesiology, CHRISTUS Children's, San Antonio, USA

**Keywords:** pediatric, dega pelvic osteotomy, hip surgery, regional anesthesia, erector spinae block

## Abstract

The erector spinae plane (ESP) block, initially designed for thoracic analgesia, has evolved into a versatile regional anesthesia technique with literature support for success in numerous contexts. In this case report, we highlight the successful application of ESP to provide postoperative analgesia for pediatric Dega osteotomy involving both the femoral head and acetabulum, in a patient with numerous neurological comorbidities that would have weighed against some more traditional regional anesthesia techniques. This case further highlights the versatility of ESP, demonstrating its use in blocking lumbar nerve roots in a pediatric patient with complex neurological challenges.

## Introduction

Regional anesthesia is useful for pain management in orthopedic surgery, including pediatric hip surgery. Innervation of the affected anatomical sites including the femoral head, acetabulum, and anterior hip comes from the proximal branches of the femoral and obturator nerves, arising from lumbar roots 2-4 (L2-L4) [1]. Several options exist for pain control in hip surgery including the fascia iliaca compartment block (FICB), femoral nerve block (FNB), paravertebral block (PVB), lumbar plexus block (LPB), quadratus lumborum block (QLB), erector spinae plane (ESP) block and the pericapsular nerve group (PENG) block [2]. The ESP block is a technique that anesthetizes nerve roots where local anesthetic solution spreads between muscle layers [3], providing analgesia to the anterior thoracic wall, abdominal wall, and parts of the hip. Several case reports document thoracic analgesia with ESP in adults and children [4], including hip surgery in adults [5], while only a few cases have been documented for lumbar spine surgery [6]. There appear to be few pediatric case reports of ESP for hip and femoral fracture surgeries [7,8]. Here, we describe our experience using an ESP block for a hip varus derotational (i.e., Dega) osteotomy in a six-year-old patient with congenital hydrocephalus and a significant neurological history. The patient’s parents provided written consent to publish this case report.

## Case presentation

**Figure 1:**
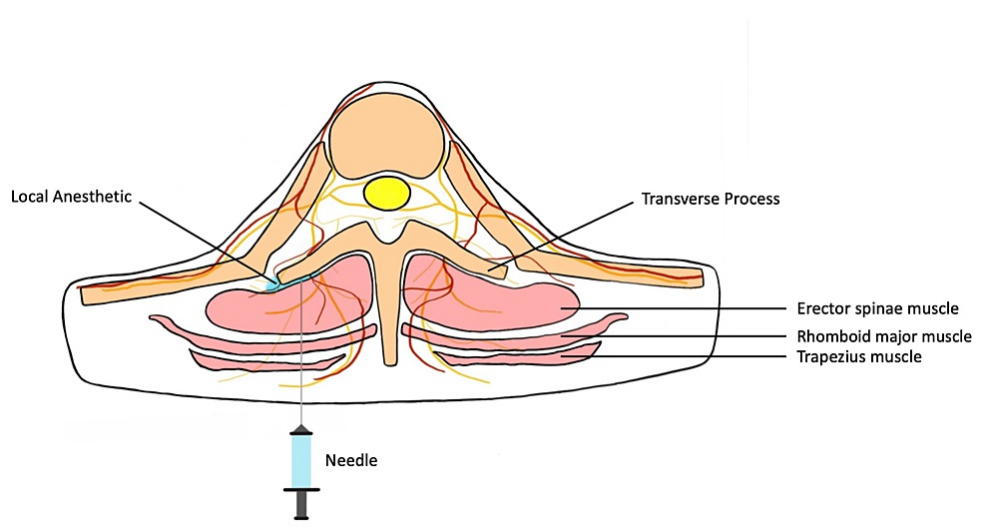
Anatomy of the different components of the erector spinae block Pearl M. Huynh provided and labeled the image

A six-year-old female (American Society of Anesthesiologists physical status class III, height 120.3 cm, weight 25.5 kg) with left spastic hip subluxation presented for a Dega osteotomy involving both pelvic and femoral osteotomies. Past medical history included prematurity (born at 29 weeks), intraventricular hemorrhage, congenital hydrocephalus status post ventriculoperitoneal shunt placement, seizures, infantile spastic cerebral palsy, right hemiplegia, bilateral optic atrophy, hearing loss, and developmental disability. Due to her significant neurologic history and congenital hydrocephalus, we decided against the placement of an epidural catheter, as well as a paravertebral catheter, due to the risk of migration into the epidural space [9]. We discussed pain management options with the patient’s mother, and she provided consent for an ESP block (Figure 1) followed by catheter placement for postoperative analgesia.

**Figure 2:**
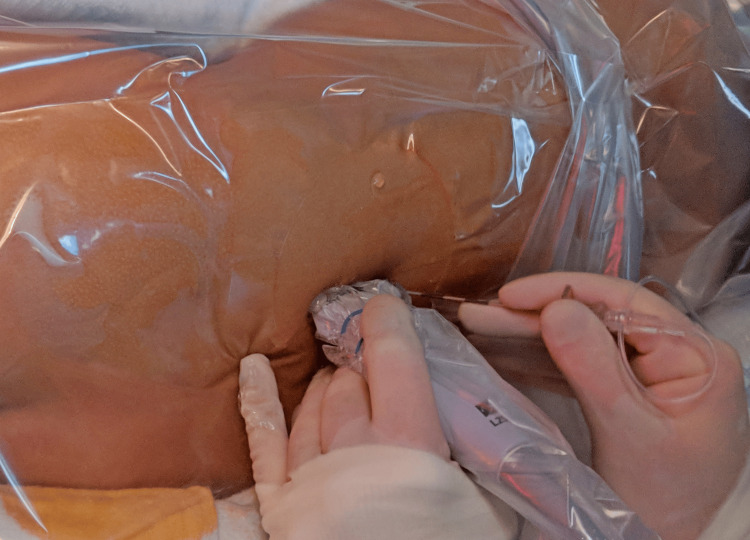
Patient in right lateral decubitus position with an ultrasound probe in parasagittal position to the left of patient’s spine

After an uneventful induction with sevoflurane, an intravenous catheter was placed. Propofol and fentanyl were administered prior to endotracheal intubation. Then the patient was placed in the right lateral decubitus position (Figure 2).

**Figure 3:**
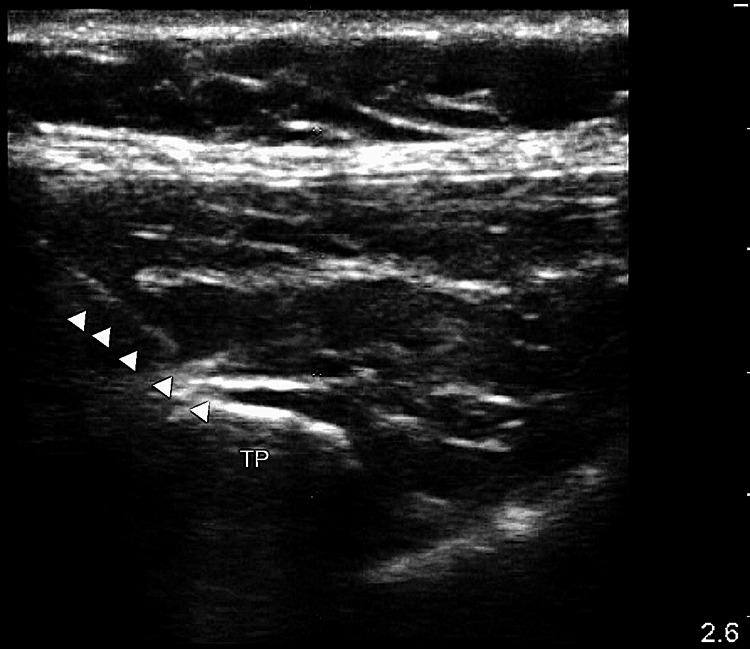
Left L2 transverse process, arrows pointing toward Tuohy needle pre-injection

**Figure 4:**
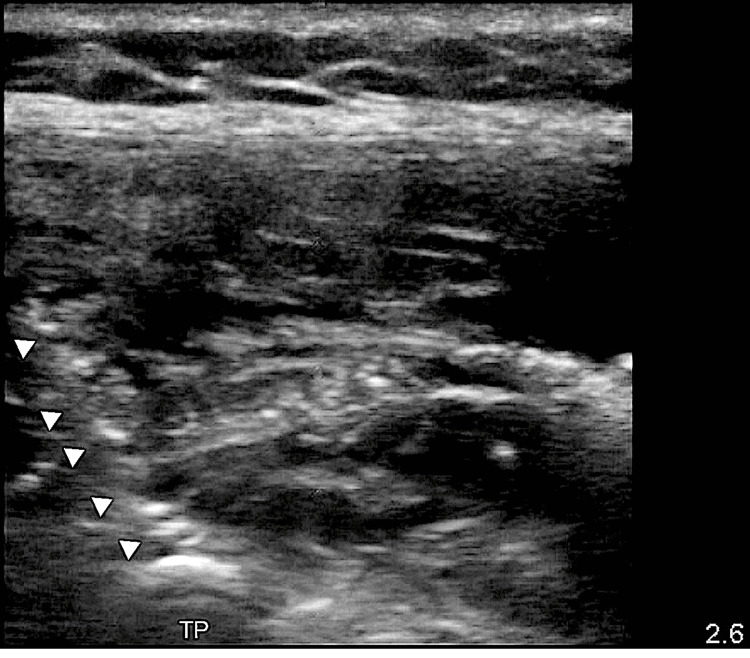
Post-injection, left L2 transverse process, arrows pointing toward the catheter

We decided to block at L2 with the needle approaching from the cranial to the caudal direction to provide both local anesthetic flow and caudad catheter placement to target the L2-L4 roots. Using ultrasound, we identified the left 12th rib by the absence of another inferior rib. Keeping a parasagittal view, we followed the rib to the transverse vertebral process. Then, we counted vertebral transverse processes caudally to find L2. We contacted the transverse process with a 17G Touhy needle, withdrew it to be superficial to the vertebra but deep to the erector spinae muscle (Figure 3), and injected 10 cc of 0.25% bupivacaine with 1 mg of dexamethasone and 1:200,000 epinephrine. We then threaded an 18G epidural catheter 4 centimeters into the space (Figure 4).

Intraoperatively, the patient received 100 µg of IV fentanyl and 15 mg of IV ketorolac. In the initial post-anesthesia care unit measurement her Face, Legs, Activity, Cry, Consolability (FLACC) scale pain level was 2, so 382.5 mg of intravenous acetaminophen was administered, and the patient was discharged to the floor for recovery.

For the first 24 hours, the patient’s pain appeared to be well controlled with FLACC scores ranging between 0 and 4. Her postoperative pain management plan consisted of the following: 12.75 mg ketorolac every six hours, 5 cc per hour of 0.1% ropivacaine via the initial erector spinae catheter, oxycodone as needed, intravenous morphine as needed for breakthrough pain, and diazepam as needed for muscle spasms. On postoperative day 0, she received 1.3 mg of oxycodone and 2.5 mg of intravenous morphine. Her pain was still well controlled on postoperative day 1 with FLACC scores ranging from 0-4, except for during one diaper change 29 hours after the block, when her FLACC score was 8. She received 2 mg of intravenous morphine. Six hours later, she received morphine again in response to pain associated with another diaper change.

In sum, on postoperative day 1, the patient received 4.5 mg of intravenous morphine and 2.5 mg of diazepam. On postoperative day 2, her FLACC score was 0-3, and she received 2 mg of intravenous morphine and 2.5 mg of diazepam. On postoperative day 3, her FLACC score was 0-2, and she received 5 mg of diazepam, ketorolac was discontinued, and no narcotics were administered. With well-controlled pain, her erector spinae catheter was removed on postoperative day 3. On postoperative day 4, she was discharged to a physical rehabilitation center. There were no respiratory, neurological, or other adverse events.

## Discussion

Given this patient’s complex medical history, we prioritized the inclusion of regional analgesia in order to permit reduced administration of opioids. However, a more traditional regional anesthesia technique such as the paravertebral approach may have been problematic in her case due to her significant neurological history associated with cerebral palsy and developmental disability. The relatively new ESP technique allowed us to provide safer postoperative analgesia for this patient in a way that met these needs. Originally described for mid- and upper thoracic analgesia, the ESP technique has also been shown to be effective for hip surgery when the lumbar nerve roots are implicated [5]. A peripheral nerve catheter can be placed into the targeted space to extend analgesia duration. Because the injection is superficial to the transverse vertebral process, there is theoretical protection of the abdominal and thoracic organs by the bone, which also may protect against accidental neuraxial injection that has been described in paravertebral blocks even when guided by ultrasound [10]. ESP provides effective analgesia and has been seen to result in high patient satisfaction [6]. 

An advantage of ESP for Dega osteotomy is that often these patients have further lower limb or femoral neck deformities, and the Dega is completed with a femoral osteotomy. An ESP can cover the sensory and motor portion of the lateral femoral cutaneous nerve, the femoral nerve, and the obturator with a single catheter covering L2 and L3. The ESP block was originally designed for thoracic analgesia but has yielded positive outcomes in a variety of surgical procedures. The use of ESP in this case, with catheter placement for extended analgesia, showcased its potential benefits in providing well-controlled postoperative pain management. The multi-level effects of ESP from a single injection and its potential coverage along the spine make it a versatile option for regional anesthesia, although further research is needed to confirm its benefits and safety.

Our experience and the literature support multi-level effects from a single injection [3,4], which can provide adequate analgesia of the anterior hip. Low thoracic ESPs, for example, have been used for abdominal surgeries [11-13]. The erector spinae muscles travel in both single and multiple vertebrae levels from the cranium to the sacrum, creating a long, contiguous fascial plane [3]. This allows for multi-level local anesthesia to spread, as seen both in cadaver studies and clinically [3], possibly primarily involving the dorsal rami of the spinal nerve roots [3,4]. If the ESP block does have a limited effect on the ventral rami, then motor weakness could be reduced or spared, creating an exciting new possibility in regional anesthesia, especially for lower extremity procedures. Further research is needed to confirm any hypothesized protective effect of ESP on lower extremity motor strength.

Given the extent of the relevant fascial plane, the ESP block may prove useful anywhere along the thoracic and lumbar spine, making it a particularly versatile option. However, the ESP block may not cover all nerves relevant to the hip. The sciatic nerve derives from both lumbar and sacral roots. Its sacral roots may not be affected by the ESP block because there is no contiguous fascial plane for local anesthesia to follow to the lower sacral roots. Its dorsal sacral nerve roots arise under the gluteus muscles, and the erector spinae muscles and thoracodorsal fascia do not pass inferior to the gluteus muscles. The ESP block is therefore unlikely to be able to provide reliably complete surgical anesthesia for hip procedures. Nevertheless, the degree of local anesthetic spread may allow the ESP block to cover several dermatomes at once, and catheters also can be placed for a longer duration, as in this case, ensuring high-quality postoperative pain management.

## Conclusions

This case report highlights the successful application of the erector spinae plane (ESP) block in providing analgesia for pediatric Dega osteotomy, demonstrating its versatility in blocking lumbar nerve roots. Overall, the ESP block represents a valuable addition to the regional anesthesia armamentarium, offering an alternative for lower extremity procedures in pediatric patients with complex neurological challenges.

## References

[1] Birnbaum K, Prescher A, Hessler S, Heller KD (1997). The sensory innervation of the hip joint--an anatomical study. Surg Radiol Anat.

[2] Dangle Dangle, J J, Kukreja P, Kalagara H (2020). Review of current practices of peripheral nerve blocks for hip fracture and surgery. Curr Anesthesiol Rep.

[3] Forero M, Adhikary SD, Lopez H, Tsui C, Chin KJ (2016). The erector spinae plane block: a novel analgesic technique in thoracic neuropathic pain. Reg Anesth Pain Med.

[4] Hernandez MA, Palazzi L, Lapalma J, Forero M, Chin KJ (2018). Erector spinae plane block for surgery of the posterior thoracic wall in a pediatric patient. Reg Anesth Pain Med.

[5] Tulgar S, Senturk O (2018). Ultrasound guided erector spinae plane block at L-4 transverse process level provides effective postoperative analgesia for total hip arthroplasty. J Clin Anesth.

[6] Oh SK, Lim BG, Won YJ, Lee DK, Kim SS (2022). Analgesic efficacy of erector spinae plane block in lumbar spine surgery: a systematic review and meta-analysis. J Clin Anesth.

[7] Elkoundi A, Bentalha A, Kettani SE, Mosadik A, Koraichi AE (2019). Erector spinae plane block for pediatric hip surgery -a case report. Korean J Anesthesiol.

[8] Balaban O, Koçulu R, Aydın T (2019). Ultrasound-guided lumbar erector spinae plane block for postoperative analgesia in femur fracture: a pediatric case report. Cureus.

[9] Visoiu M, Scholz S (2019). Sonographic documentation of dislodged paravertebral catheter into the epidural space in a young infant. Paediatr Anaesth.

[10] Albi-Feldzer A, Duceau B, Nguessom W, Jayr C (2016). A severe complication after ultrasound-guided thoracic paravertebral block for breast cancer surgery: total spinal anaesthesia: A case report. Eur J Anaesthesiol.

[11] Chin KJ, McDonnell JG, Carvalho B, Sharkey A, Pawa A, Gadsden J (2017). Essentials of our current understanding: abdominal wall blocks. Reg Anesth Pain Med.

[12] Chin KJ, Malhas L, Perlas A (2017). The erector spinae plane block provides visceral abdominal analgesia in bariatric surgery: a report of 3 cases. Reg Anesth Pain Med.

[13] Restrepo-Garces CE, Chin KJ, Suarez P, Diaz A (2017). Bilateral continuous erector spinae plane block contributes to effective postoperative analgesia after major open abdominal surgery: a case report. A A Case Rep.

